# Muscle Tissue as a Surrogate for In Vitro Drug Release Testing of Parenteral Depot Microspheres

**DOI:** 10.1208/s12249-021-01965-4

**Published:** 2021-03-29

**Authors:** Jan Kozak, Miloslava Rabiskova, Alf Lamprecht

**Affiliations:** 1 Department of Pharmaceutics, Institute of Pharmacy, University of Bonn, Gerhard-Domagk-Straße 3, 53121 Bonn, Germany; 2 Department of Pharmaceutical Technology, Faculty of Pharmacy, Charles University, Akademika Heyrovskeho 1203/8, 500 05 Hradec Kralove, Czech Republic

**Keywords:** in vitro release,, biorelevant,, depot microspheres,, intramuscular,, PLGA

## Abstract

Despite the importance of drug release testing of parenteral depot formulations, the current *in vitro* methods still require ameliorations in biorelevance. We have investigated here the use of muscle tissue components to better mimic the intramuscular administration. For convenient handling, muscle tissue was used in form of a freeze-dried powder, and a reproducible process of incorporation of tested microspheres to an assembly of muscle tissue of standardized dimensions was successfully developed. Microspheres were prepared from various grades of poly(lactic-co-glycolic acid) (PLGA) or ethyl cellulose, entrapping flurbiprofen, lidocaine, or risperidone. The deposition of microspheres in the muscle tissue or addition of only isolated lipids into the medium accelerated the release rate of all model drugs from microspheres prepared from ester-terminated PLGA grades and ethyl cellulose, however, not from the acid-terminated PLGA grades. The addition of lipids into the release medium increased the solubility of all model drugs; nonetheless, also interactions of the lipids with the polymer matrix (ad- and absorption) might be responsible for the faster drug release. As the *in vivo* drug release from implants is also often faster than in simple buffers *in vitro*, these findings suggest that interactions with the tissue lipids may play an important role in these still unexplained observations.

**Supplementary Information:**

The online version contains supplementary material available at 10.1208/s12249-021-01965-4.

## Introduction

The drug release rate from implantable formulations is a critical parameter of their therapeutic effectiveness. However, the release rate and duration obtained by the current *in vitro* release testing methods often strongly differ from the subsequent results of animal and human trials as the oversimplified *in vitro* conditions fail to mimic the complex environment of the tissue. The factors responsible for these differences are yet mostly unknown or only hypothetical. An ideal “biorelevant” *in vitro* method would specifically consider all factors affecting drug release *in vivo*. The urgent need for biorelevant *in vitro* methods has been repeatedly emphasized (1–4), as achieving a more accurate simulation can reduce patients’ risk of receiving an inadequate daily dose, accelerate formulation development, and decrease the number of animal experiments.

The biorelevant dissolution has been for a long time vividly investigated topic in the field of oral formulations. Enhancing simple buffers used as dissolution media by additional physiological parameters (e.g., surfactants, enzymes, concomitant food intake, biphasic dissolution, etc.) has been in many cases shown to provide better *in vitro*-*in vivo* correlations (5–7). In contrast, methods for the parenteral formulations are nowadays still at the stage of simple buffered media. Furthermore, due to the lack of standardization and no specific pharmacopeia apparatus, the setups largely vary between the research groups: either simple agitated/stirred vials, dialysis membranes (for a physical separation of particulate systems), or flow-through cells (mostly the USP 4 apparatus, originally designed for oral formulations) are currently applied (3). Independently on the settings, they all rely on the use of simple buffered release media (mostly phosphate buffer) of physiological pH 7.4 and temperature 37°C (3, 4). Only few studies have described attempts on application of biorelevant media, either with respect to the ionic composition (8) or with additional specific components of extracellular matrix (ECM)—such as hyaluronic acid either in a dialysis model mimicking the subcutaneous tissue (9) or as a component of a simulated synovial fluid (10–12). An alternative approach is the release testing in hydrogels which are supposed to simulate the gel-like physical nature of the ECM (13–19); however, the hydrogel-forming polymers used, such as agar or agarose, do not resemble the specific chemical composition of the tissue. Electrostatic binding interactions between the released drug and the components of the ECM can occur since the collagen is at the physiological pH positively charged and the hyaluronic acid and chondroitin sulphate are negatively charged (9, 20).

The drug release from implants is often faster *in vivo* than during the *in vitro* testing in simple buffers (21–26). In the case of biodegradable polyesters such as poly(lactic-co-glycolic acid) (PLGA), this is being explained by faster hydrolytic degradation of the polymer and erosion due to the enzymes present in the living tissue, despite the fact that the effect of the enzymes is inconclusive and many studies are supporting nonenzymatic hydrolysis (22, 23, 27). Therefore, attempts should be made to investigate the influence of further components present in the tissue environment to better understand the complex *in vivo* situation. Amongst the diverse components in the tissue which can affect the structural integrity of the implanted materials are the lipids (28, 29). The locations of intramuscular (i.m.) or subcutaneous (s.c.) administration—the muscle and the adipose tissue—are heavily involved in the turnover of lipids and fatty acids: there is a constant “flux” of lipids and fatty acids through the blood capillaries and the interstitium either as a source of energy (muscle) or storage and release for other cells (adipose tissue). Needless to say, also the membranes of cells and extracellular vesicles are formed by a lipid bilayer. Therefore, the lipids and the fatty acids may likely either directly interact (e.g. ad-/absorption) with the i.m./s.c. implanted microspheres or contribute to drug partitioning and so influence the drug release rate.

Excised animal tissues are commonly used for the *in vitro* testing of pharmaceuticals, as they provide morphology and chemical composition equivalent to the intended site of administration: e.g., human/porcine skin for topical formulations and transdermal patches (30, 31), or porcine eyes for ophthalmic formulations (32–34). Analogically, we have investigated here for the first time the possibility of application of excised porcine muscle tissue as an *in vitro* model simulation of the intramuscular environment for the testing of depot microspheres.

The muscle tissue was used in form of freeze-dried powder for advantageous handling and reproducibility compared with the bulk muscle tissue. The tested microspheres were prepared out of ethyl cellulose or various grades of PLGA. Several studies have reported faster risperidone release from depot PLGA microspheres *in vivo* than *in vitro* (26, 35); therefore, we have chosen the risperidone as a good candidate for encapsulation to study the influence of the investigated release testing conditions and their biorelevance. Flurbiprofen and lidocaine were also included as additional drugs for encapsulation. We have further investigated the effect of lipids isolated from the muscle tissue on drug solubility and drug release from the microspheres; and in addition also a binding interaction of the model drugs with the freeze-dried muscle tissue.

## Materials and Methods

### Materials

The PLGA was obtained from Evonik (Darmstadt, Germany) under the commercial name Resomer® RG. PLGA grades of 50:50 lactic to glycolic acid ratio and of different molecular weights (Mw) and either acid- or ester-terminated were obtained: Resomer® RG 503H (acid-terminated, Mw 24 000–38 000); Resomer® RG 504 (ester-terminated, Mw 38 000–54 000); and Resomer® RG 505 (ester-terminated, Mw 54 000–69 000). Ethyl cellulose was obtained under the commercial name Ethocel^TM^ Standard 7 Premium from Dow (Midland, MI, USA). Risperidone and flurbiprofen were obtained from Acros Organics (Geel, Belgium). Lidocaine and agarose type I-B were obtained from Sigma-Aldrich. All other chemicals were of analytical grade or higher.

### Microsphere preparation

The microspheres were prepared using simple oil-in-water (o/w) emulsion solvent extraction/evaporation method as described earlier (19). The drug (flurbiprofen, lidocaine, or risperidone; 120–250 mg, depending on the drug loading) and the polymer (PLGA or ethyl cellulose) in a total amount of 1 g were dissolved in 7-ml dichloromethane and emulsified in 20 ml of 1% polyvinyl alcohol (PVA) under 750 rpm for 1 minute. The emulsion was transferred into 1 L of 0.1% PVA to allow evaporation of the dichloromethane under constant stirring (300 rpm). The microspheres were washed, collected on a filter, and vacuum-dried for 24 hours. After drying, the particles were sieved through 150-μm mesh size to remove agglomerates and were used either directly after preparation or stored at 4°C until used (stored not longer than 1 week). The size of the microspheres was measured using a laser diffraction particle size analyzer LA-960 (HORIBA, Kyoto, Japan). The median particle size is given in the following sections as a parameter D50.

To determine the drug loading, 20.0 mg of the microspheres was dissolved in 2 ml of acetonitrile and afterwards filled up to 25.0 ml using either 0.1-M HCl (in case of risperidone and lidocaine) or 0.1-M NaOH (in case of flurbiprofen). The drugs were analyzed using HPLC methods described below.

### The simulated muscle setup

As recognized during initial trials, direct injection of the tested microspheres into a bulk excised muscle tissue would result in several problems:

1. Unknown localization of all “administered” microspheres and consequently problematic localization of the released drug.
2. Destructive sampling (pieces of muscle tissue as samples would be necessary) and formation of a concentration gradient of the released drug of decreasing concentration with increasing distance from the dosage form. In addition, the usual “media replacement” with the equivalent volume of fresh medium very easily applicable in case of liquid release media would be very complicated in case of a muscle tissue.
3. Risk of incomplete drug recovery from muscle tissue.
4. Microbial instability of the tissue over the long time period at 37°C (several weeks can be expected in case of implantable microspheres).

To overcome the abovementioned problems, novel approach was developed here. The muscle tissue was freeze-dried and subsequently pulverized for better handling and reproducibility. The tested microspheres were then incorporated together with the freeze-dried muscle tissue powder into a small assembly held together by agarose hydrogel (procedure described in detail below), enabling that the microspheres are in direct contact with the muscle tissue, and at the same time, the small size of the assembly allows its placing into aqueous buffered release medium (simulating blood compartment), from which the samples can be conveniently taken and analyzed using conventional techniques such as HPLC. The aqueous release medium can be easily replaced in contrast to bulk muscle tissue providing advantage of nondestructive sampling. The agarose was selected for its excellent long-term stability (36) and to mimic the gel-like nature of ECM (37–39). Although the microspheres are intended to be suspended in a vehicle before administration to provide injectability, the release rate mechanism is determined solely by the characteristics of the microspheres and the vehicle is not intended to contribute to the sustained release. Therefore, incorporation of microspheres directly in muscle tissue without vehicle will not significantly alter the results.

### Preparation of muscle tissue powder

Porcine muscle tissue (from hind limb) was bought at a local butcher, cut to smaller pieces, frozen at −30°C overnight, and freeze-dried in Steris Lyovac GT2 freeze dryer (Mentor, OH, USA). The tissue was weighed before and after freeze drying to determine the water content. The freeze-dried tissue was then pulverized in Retsch Centrifugal mill ZM1 (Haan, Germany) in two cycles of decreasing mesh size (mesh sieve size 2 mm followed by 0.5 mm).

### Incorporation of microspheres into the simulated muscle setup

**Fig. 1:**
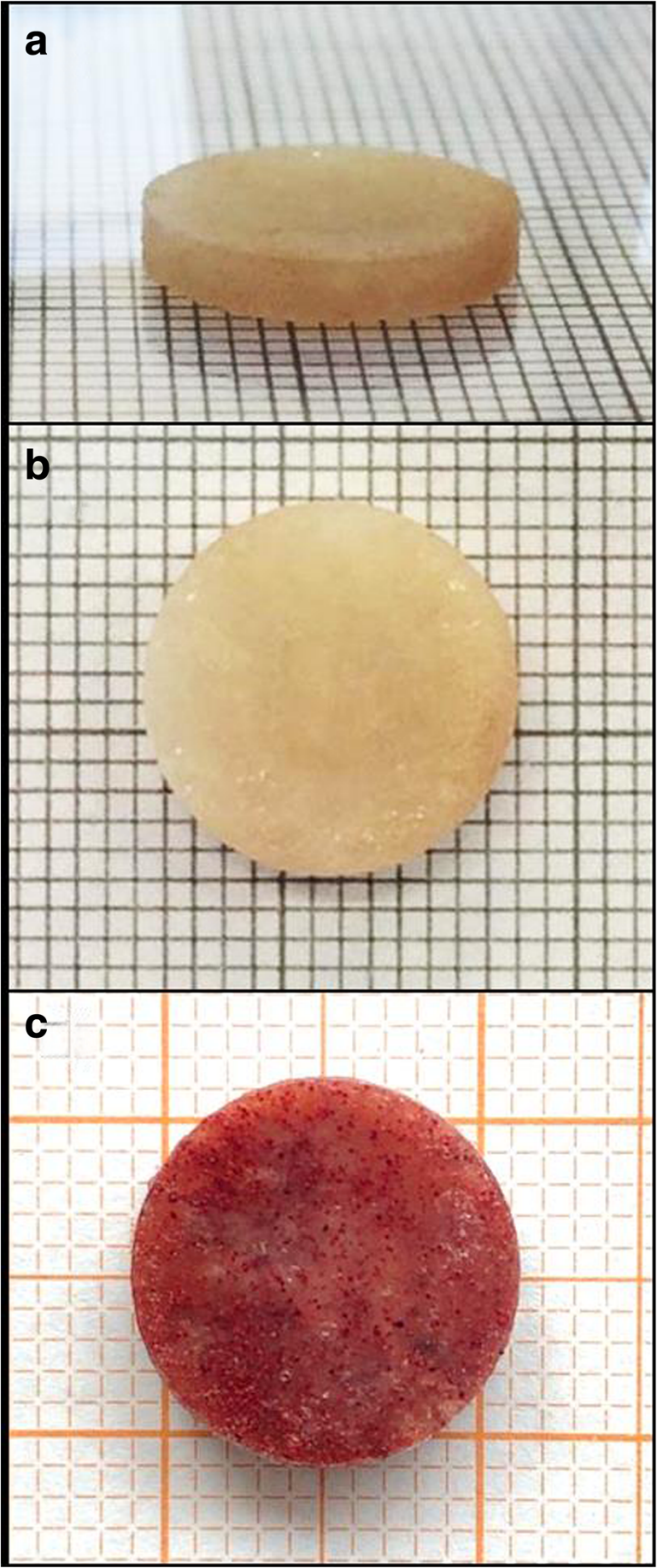
The setup of muscle tissue with incorporated microspheres held by agarose hydrogel. The preparation procedure enables standardized and reproducible dimensions and shape. **a** side view and **b** top view of muscle setup with drug-loaded microspheres. **c** Ethyl cellulose microspheres loaded with water-insoluble red dye (Sudan III) were used to visualize the distribution of microspheres

Microspheres (25.0 mg) and muscle tissue powder (50 mg) were weighed on an analytical balance and mixed using a spatula. Agarose hydrogel (2%) was used as a binding matrix for the mixture of microspheres and the freeze-dried muscle tissue powder so that the resulting assembly does not disintegrate upon placing into release medium. Importantly, not pure water, but the release medium was used for the preparation agarose hydrogel, to provide buffered conditions and pH 7.4. In order to solubilize the agarose, the suspension must be heated to 90–95°C and the sol-gel transition occurs spontaneously upon cooling below 35–37°C. Such high temperatures over 90°C even for short contact time could affect the tested microspheres (burst release or softening of PLGA); hence, the solubilized agarose was cooled to around 40°C under vigorous stirring (necessary to prevent the sol-gel transition to occur already before application). To provide a reproducible shape and dimensions of the resulting agarose gel/muscle tissue/microspheres setup, an assembly of flat punches and a die (12 mm diameter) made of stainless steel was used. Firstly, one punch was partially inserted into the die from below, to form a cavity into which the dry blend of muscle tissue powder with the microspheres was filled. The cooled 2% agarose solution (170 μl) was added using a micropipette and the second punch was quickly introduced from the top. The weight of the upper punch applied sufficient pressure to allow the (liquid) cooled agarose solution to homogenously penetrate into the blend of muscle powder and microspheres, before the sol-gel transition of agarose occurred. The added volume of the agarose solution was selected to correspond to the amount of water lost during the freeze-drying step, in order to keep the ratio of water and solids the same as in the original muscle tissue. The procedure was performed to obtain a normalized reproducible shape and dimensions—the diameter is determined by the inner diameter of the die and the height by the constant mass (weighing on an analytical balance and using a micropipette provides sufficient precision and reproducibility). The thickness of the final disc-shaped assembly was approx. 2 mm (Fig. 1). The final assembly was left standing for 5 minutes to allow complete gelation of agarose hydrogel and then transferred into 20.0 ml of release medium.

### Extraction of muscle lipids

The freeze-dried muscle powder was suspended in a mixture of chloroform/methanol in a 2:1 volume ratio and horizontally shaken for 12 hours. Afterwards, the insoluble components (predominantly the muscle proteins) were separated by filtration and the volatile solvent mixture was left to evaporate at room temperature, followed by vacuum drying for 24 hours to remove the solvent residues. The extracted lipids were subsequently emulsified in the release medium using an IKA Ultra Turrax® T 18 (Staufen, Germany). Some components of the extracted lipids (most likely lecithin and free fatty acids), as well as the 0.02% concentration of polysorbate 20 in the release medium, acted as surfactants, facilitating the emulsification and stabilizing the emulsion formed. The concentration used for drug release testing was 30 mg lipids/ml of the release medium unless stated differently.

The lipids emulsified in the release medium create a biphasic system of lipid droplets and micelles. The focus was confined on the effect on the drug release from the microspheres; therefore, the released drug was determined in both phases together. The fractions of the drug in the lipid phase and in the aqueous phase were not separately investigated, since the drug partitioning is already a step *after* the release from the microspheres and not determined by the dosage form formulation; it is only determined by the physicochemical properties of the particular drug (such as log D).

The muscle tissue powder without the extracted lipids was also kept and used to prepare the same assembly with microspheres as described in previous section, in order to test the release behavior when the lipids were not present.

### Drug release conditions

The release medium consisted of 0.1-M sodium phosphate buffer (pH = 7.4) with osmolarity adjusted to 285 mosm/ml with sodium chloride; 0.05% sodium azide was used to prevent microbial growth and 0.02% polysorbate 20 to provide sufficient wettability of the microspheres and to prevent their aggregation. Sink conditions were maintained for all drugs. The temperature of 37°C was chosen for better consistency with other studies, although the average temperature of resting muscles is between 34 and 35°C (40). The investigated release conditions were compared with the simultaneous testing of the microspheres of the same batch under current “standard conditions” by freely suspending in the release medium alone. The pH of the release medium was regularly checked during the period of release testing and adjusted in case of pH drop caused either by hydrolysis of the PLGA or the lipids. Due to the inevitable hydrolysis of the lipids to free fatty acids, the pH of the medium with 30 mg/ml of emulsified lipids slowly decreased in time (despite the buffering capacity of 0.1-M phosphate buffer) by approx. 0.1 on the pH scale in 7 days if no pH adjustment was performed. One quarter of the volume of the release medium was replaced in each sampling point to introduce medium with “fresh” lipids. The vials with the release medium with emulsified lipids were mildly manually agitated in each sampling point to prevent phase separation of the emulsion (the control vials with lipid-free medium were also agitated to eliminate the possible influence on drug release).

### Determination of drug binding on muscle tissue

Protein binding was determined in suspension of the freeze-dried muscle tissue powder in the release medium (20 mg/ml). Into 9.0 ml of the suspension, 1000 μl of stock solution of the drug (100μg/ml) was added and the suspension was incubated at 37°C for 24 hours. The drug concentration was chosen to approximately correspond to the concentrations present during the drug release. To determine the binding on the soluble proteins, the blank suspension was incubated for 24h under 37°C, centrifuged, and the drug stock solution was added to the supernatant and incubated for additional 24 hours. As a control, the drug stock solution was added to the pure release medium. All experiments were performed in triplicate. The drug concentration after incubation was determined by the HPLC methods described below. The drug recovery was calculated as percentage of the peak area in relation to the control in pure medium.

### Drug solubility

The saturation solubility (Cs) was determined in 3 ml of either pure release medium or with different concentrations of emulsified muscle lipids (3, 15, 30, and 60 mg/ml). The drug suspension was incubated at 37°C for 2 days to obtain a saturated solution in equilibrium. The undissolved particles of drug excess were filtered through a 0.2-μm pore size syringe filter, diluted, and analyzed using respective HPLC methods described below.

### Liquid chromatography analysis (HPLC)

Analyses were performed with a Waters Alliance (Waters, Milford, MA, USA) HPLC 2695, equipped with a Waters 996 photodiode array detector. All three drugs were analyzed on the C-18 column LiChrospher 100 RP 18-5μm EC (CS-Chromatographie, Merck) based on previously described methods (19). The mobile phase for flurbiprofen consisted of a mixture of acetonitrile and water buffer (1% chloroacetic acid adjusted with ammonium hydroxide on pH 3.0) in ratio 60/40; flurbiprofen was detected at 244-nm wavelength. The mobile phase for risperidone was a mixture of acetonitrile and water buffer (0.1% acid adjusted with ammonium hydroxide to pH = 3.0) in a ratio of 65/35; risperidone was detected at 273-nm wavelength. The mobile phase for lidocaine consisted of acetonitrile and 0.01-M phosphate buffer of pH = 6.5 in a ratio of 65/35. The UV detection was at 215 nm. The linearity was determined in a range between 0.5 and 50 μg/ml (for further details, see Supplementary Material).

An addition of an equivalent volume of acetonitrile to the samples of the muscle setup or lipid-containing medium resulted in a complete drug recovery for the HPLC analysis.

### Contact angle (wettability)

The films for the contact angle measurements with 8% drug loading were prepared by a solvent casting method by dissolving the drug and respective polymer in dichloromethane, casting on nonadhesive Petri dish and evaporating. The contact angle was measured using Drop Shape Analyzer EasyDrop (Krüss, Hamburg, Germany), as a sessile drop on the film surface (*n* = 6).

### Differential scanning calorimetry (DSC)

The instrument DSC 2 (Mettler Toledo, Giessen, Germany) with nitrogen as a cooling gas was used. The heating-cooling-heating cycle was: +25°C/−20°C/+60°C/−20°C/+60°C at a rate of 10K/min. The samples were analyzed at hydrated state as taken from the release medium in aluminum pans with nonpierced lid and weighted after each run to assure no water was lost during the heating.

## Results

### Binding interactions with the muscle tissue

We found that flurbiprofen and lidocaine partially bind on the structural components of the muscle tissue. When the stock solution of flurbiprofen was added to the release medium with 20 mg/ml suspended muscle tissue powder, only 79.2 ± 0.9% recovery was determined in relation to the simultaneously performed blank (without muscle tissue). The same procedure with lidocaine showed only 91.3 ± 1.1% recovery; in the case of risperidone, complete recovery (99.8 ± 0.5%) was determined. However, complete drug recovery of all 3 model drugs was obtained when the drug solution was added to the supernatant (soluble components of the muscle tissue). This suggests that the flurbiprofen and lidocaine bound to the structural insoluble components of the tissue.

### Effect of muscle lipids on drug solubility

**Fig. 2:**
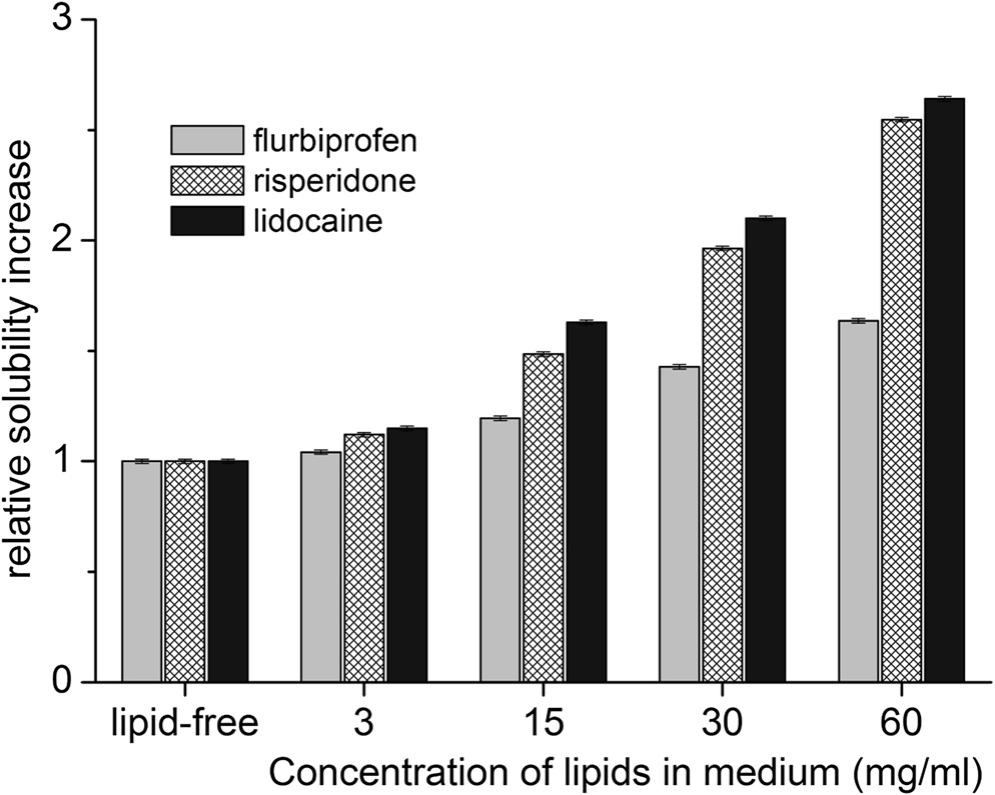
Relative increase in saturation solubility (the solubility in lipid-free medium is set as a reference value of 1) of the model drugs with increasing concentration of the lipids in the release medium

With increasing concentration of emulsified muscle lipids in the release medium, the saturation solubility of all three model drugs gradually increased (Fig. 2). For instance, with 30 mg/ml of lipids, the saturation solubility (Cs) of flurbiprofen increased from 7.92 ± 0.05 mg/ml in the lipid-free release medium to 11.55 ± 0.09 mg/ml, the Cs of lidocaine from 9.94 ± 0.06 to 20.87 ± 0.18 mg/ml, and the Cs of risperidone from 0.32 ± 0.01 to 0.61 ± 0.01 mg/ml.

### Drug release under investigated conditions

The muscle setup (Fig. 1) remained mechanically stable over the whole period of the release testing without disintegrating in the release medium. The sodium azide efficiently prevented microbial instability over the period of drug release as no organoleptic signs of microbial deterioration were apparent. Only the color of the muscle tissue changed after the first day in the medium from the original light pink to whitish.

**Fig. 3:**
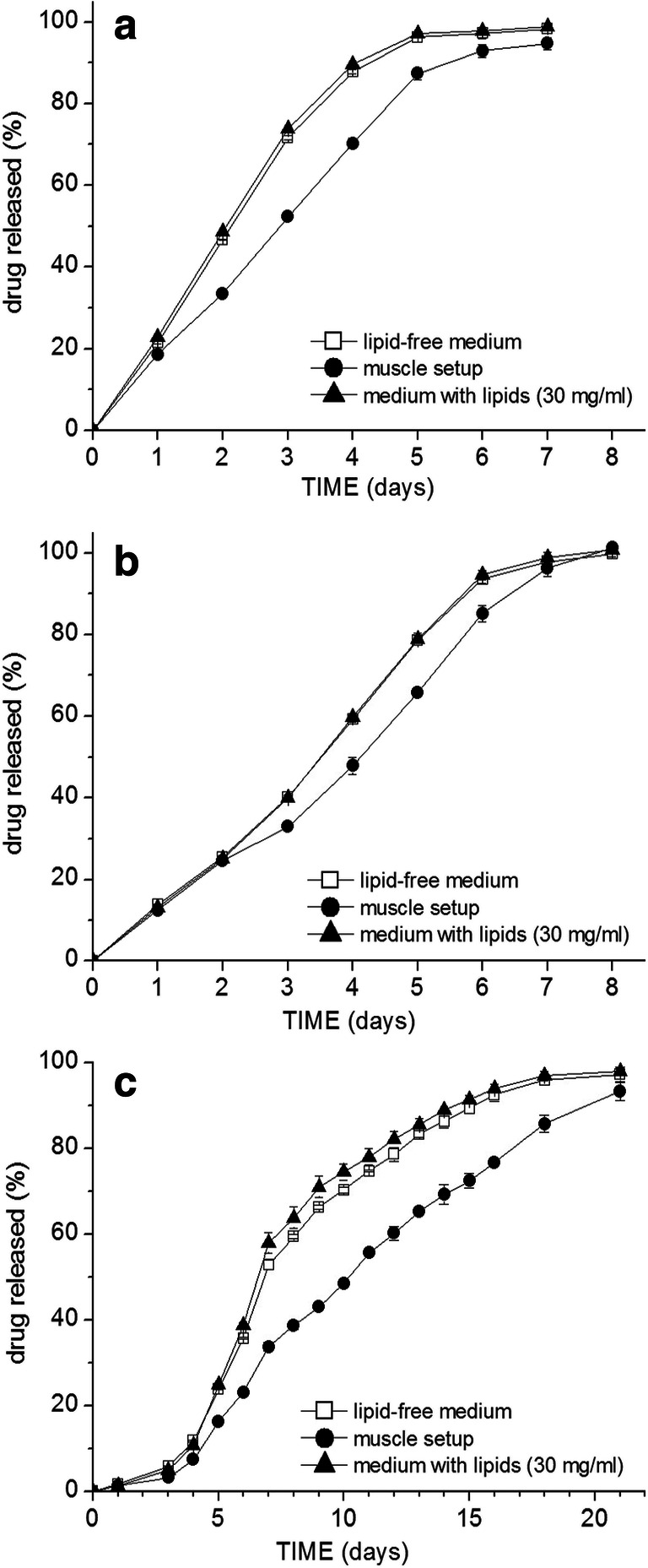
Drug release profiles of Resomer® 503 H microsphere formulations loaded with **a** 6.5% flurbiprofen (D50 = 82 μm), **b** 6.9% lidocaine (D50 = 86 μm), **c** 7.4% risperidone (D50 = 81 μm) under the different testing conditions (notice the different scaling of the X axis)

The release of the three drugs from the microspheres prepared from the acid-terminated Resomer® grades (502H, 503H, and 504H) was in all cases slower in the muscle setup than when they were freely suspended in the release medium. The release profiles from the 503H grade microspheres are given as an example in Fig. 3. Differences were generally more pronounced in the case of risperidone than flurbiprofen and lidocaine. The addition of lipids into the medium (30 mg/ml) did not have any impact on the lidocaine release from the acid-terminated grades and the release of flurbiprofen and risperidone was only marginally faster (Fig. 3).

**Fig. 4:**
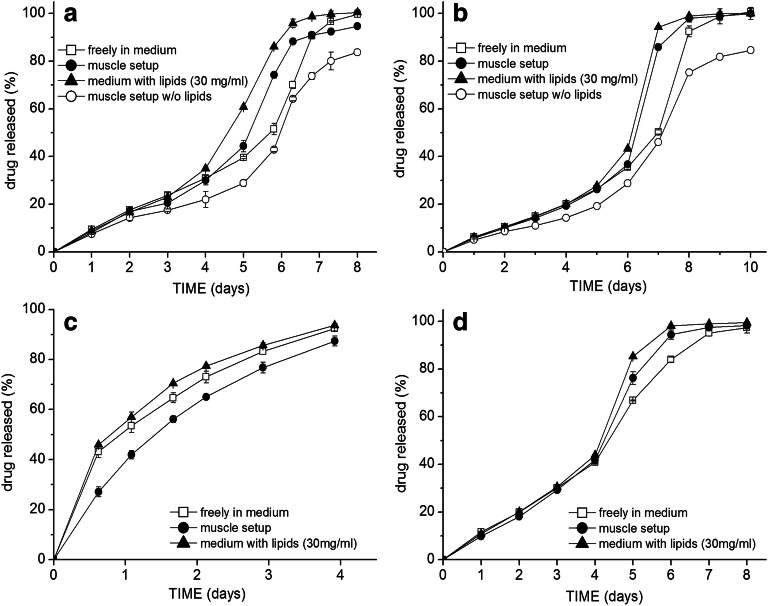
Drug release profiles of flurbiprofen under different testing conditions from microspheres of ester-terminated PLGA grades. 505 grade microspheres with lower drug loading and either different sizes: **a** 7.2% flurbiprofen, D50 = 86 μm or **b** 7.4% flurbiprofen, D50 = 112 μm; **c** higher flurbiprofen loading of 15.1% (D50 = 84μm); **d** 504 grade (6.8% flurbiprofen, D50 = 78 μm)

However, different release behaviors were observed in the case of the ester-terminated 505 grade. When the flurbiprofen-loaded Resomer® 505 grade particles were tested, the release was faster in the muscle setup (Fig. 4a): the difference was most prominent at the 5.8-day time point with 74.1 vs. 51.5% released in the muscle setup vs. in pure medium, respectively, and at the time point of 6.3 days, 88.2 vs. 70.0%. From the further results in Fig. 4a, it is apparent that with the addition (emulsification) of only the isolated muscle lipids, even stronger acceleration of flurbiprofen release occurred. On the contrary, if the particles were incorporated in the assembly made out of muscle tissue powder after the removal of the lipids, the release was even slower than in the release medium alone. This clearly shows the determining effect of the lipids on the acceleration of the flurbiprofen release. The *T*_g_ of the particles after 5 days (approx. the time point when the release profiles started to differentiate) in the release medium either with or without lipids was determined (in a hydrated state)—in both cases, the *T*_g_ was around 21°C, indicating no significant plasticizing effect of the lipids.

This effect on drug release was similar for different average particle sizes with similar flurbiprofen loading (compare Fig. 4a and b). In the initial phase, the release curves were overlapping, indicating no retarding impact of the additional drug diffusion step through the muscle tissue.

The same accelerating trend was observed also in the case of the Resomer® 504 formulation (lower Mw than the 505) (Fig. 4d): on day 5, there was already 85.3% flurbiprofen released in the medium with lipids, 76.2% released in the muscle setup while only 66.7% in release medium alone; on day 6, then 98.1 vs. 94.4 vs. 83.9%, respectively.

With a higher flurbiprofen drug loading in 505 microspheres (Fig. 4c), the drug release was very rapid and complete within 4 days, the effect of lipids was less pronounced, but the release in case of the muscle setup (despite lipids were not removed) was retarded.

**Fig. 5:**
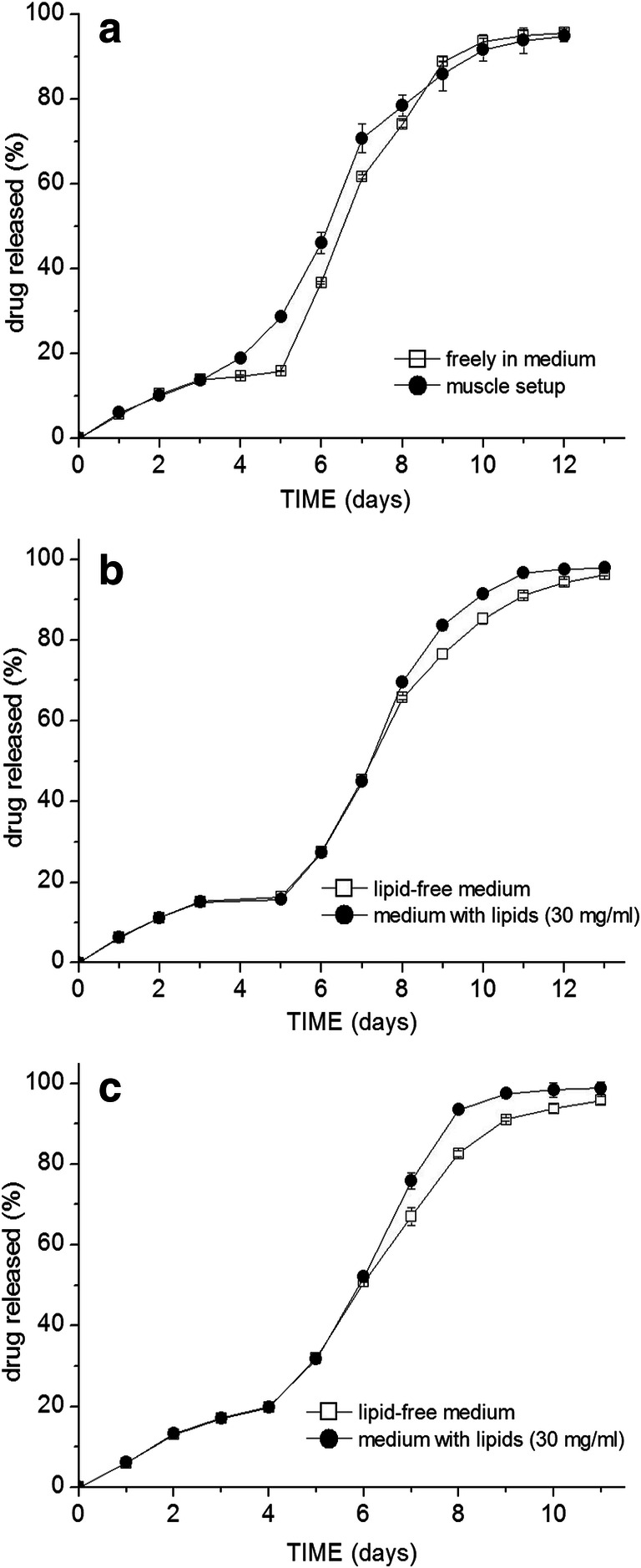
Comparison of lidocaine release from Resomer® 505 microspheres between pure release medium and **a** muscle setup (7.1% lidocaine, D50 = 85 μm), or with different drug loadings: **b** (7.3%; D50 = 83 μm) and **c** (15.8%; D50 = 85 μm) in medium with isolated muscle lipids (30 mg/ml)

The release of lidocaine from the 505 grade particles was also accelerated. Interestingly, the release in the muscle setup was accelerated between days 4 and 7 (Fig. 5a), while the emulsified lipids accelerated the release only in the final days and the release curves in the preceding days were overlapping (Fig. 5b and c).

**Fig. 6:**
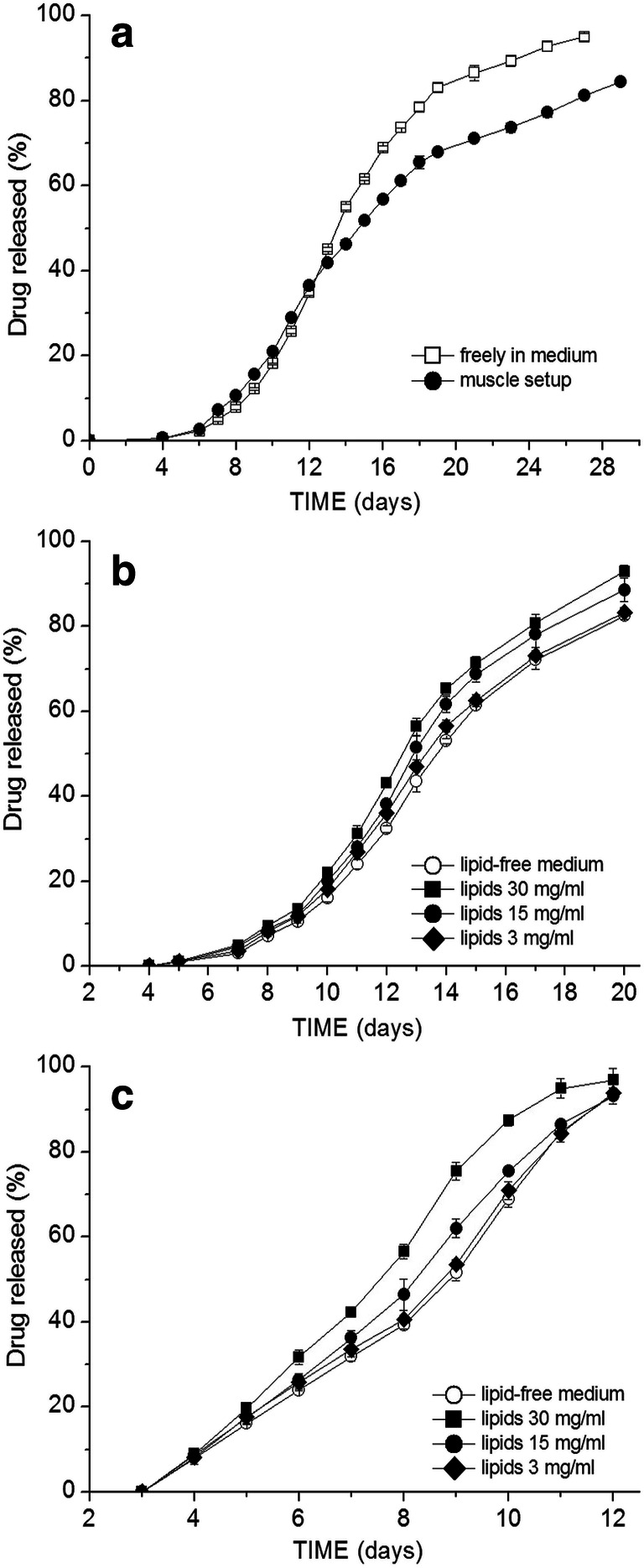
Comparison of risperidone release from Resomer® 505 microspheres between pure release medium and **a** muscle setup (8.5% risperidone, D50 = 78 μm), or with different drug loading: **b** (8.9%; D50 = 82 μm) and **c** (16.1%; D50 = 79 μm) with addition of different concentrations of isolated muscle lipids

When the risperidone loaded 505 microspheres were tested in the muscle setup, there was also marginally faster risperidone release observed between days 7 and 12 (Fig. 6a), but not as prominent as in the case of flurbiprofen or lidocaine. From day 13 onwards, the released amount was, on the contrary, lower in the case of the muscle setup.

The effect of different lipid concentrations in the medium (3, 15, and 30 mg/ml) was tested on two 505 formulations with different risperidone loadings. The release rate increased with increasing concentration of emulsified lipids in the release medium (Fig. 6b and c). The trend was similar for both drug loadings; however, the degree of the difference from the lipid-free medium was more pronounced in the case of the formulation with higher drug loading. The lipid concentration of 3 mg/ml was the lowest where any significant effect on release could be observed.

The contact angle on the surface of the Resomer® 505 films loaded with 8% of either of the model drugs was measured to determine a possible effect of the lipids on the wettability of the medium on the hydrophobic surface. There was no difference between the lipid-free medium (37.3 ± 3.8°) and the medium with 30 mg/ml of lipids (36.8 ± 3.5°). The particles in the lipid-free medium were already well wetted given by the presence of 0.02% polysorbate 20, sank, did not float on the surface of the medium, did not agglomerate, and remained individually suspended; hence, it is unlikely that a change in wettability contributed to the faster release.

**Fig. 7:**
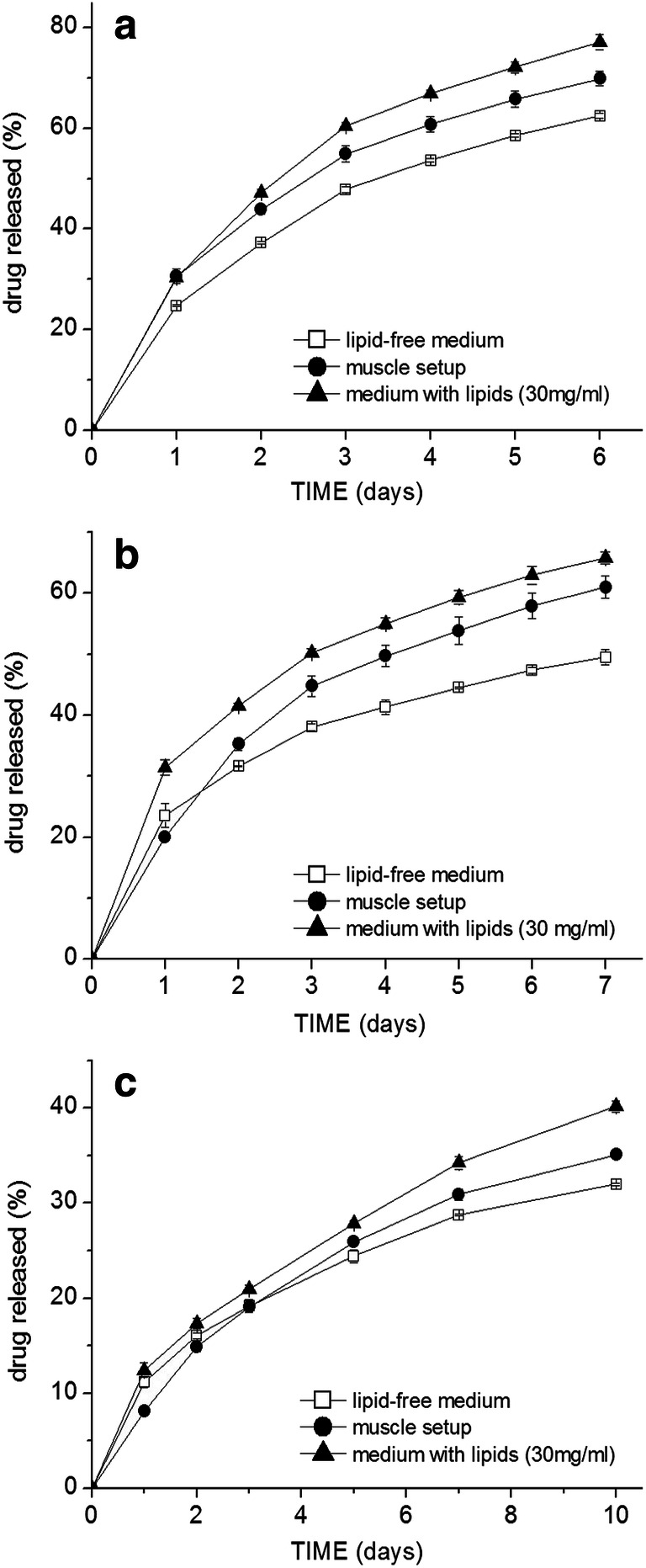
Release profiles from the ethyl cellulose microspheres loaded with either **a** 8.5% flurbiprofen (D50 = 70 μm), **b** 8.8% lidocaine (D50 = 71 μm), **c** 9.1% risperidone (D50 = 77 μm)

The lipids in the release medium as well as the incorporation into the simulated muscle setup also significantly impacted—accelerated—the drug release from the ethyl cellulose formulations. The release of all three model drugs from the ethyl cellulose microspheres followed the same trend, slowest release in the pure medium, fastest in the medium with emulsified lipids, and the release curve obtained in the muscle setup was in between (Fig. 7), with an exception in case of the first day time point on the release curves of lidocaine and risperidone with the lowest released amount in the muscle setup. The presence of lipids accelerated the release already from the first day, whereas in the case of the 505 PLGA grade, the difference was prominent only in the final stages. Due to the diffusion-controlled release from ethyl cellulose matrix, the release rate gradually decreased; however, as apparent from the slope of the release curves, this decrease in the release rate was less prominent in the muscle setup and in the presence of lipids than in the pure medium—resulting in the overall faster release. This effect was at most pronounced on the least soluble risperidone after the first week of release.

## Discussion

Muscle tissue in its freeze-dried pulverized form offered a worthwhile approach in the development of a first model simulation of intramuscular environment for drug release testing. The investigated factors in most cases affected the drug release and can therefore provide additional information about the factors influencing the release *in vivo* than testing in simple buffers.

Flurbiprofen and lidocaine are known to bind strongly on plasma proteins (41–43) and a binding tendency towards the muscle tissue was also observed in our study. Such binding interactions of the released drug on the structural proteins of interstitium (e.g., collagen or elastin) might occur *in vivo* and should be also considered in development of biorelevant methods. However, the binding interaction was not responsible for the observed slower drug release from the acid-terminated PLGA grades in the muscle setup than in pure medium, because in the case of the ester-terminated grades and the ethyl cellulose, the release in the muscle setup was, on the contrary, faster than in pure medium. Furthermore, the release profiles between the muscle setup and pure medium differed at most for risperidone despite no binding of risperidone was determined. The slower release in the muscle setup can be more likely explained by swelling restriction of the particles tightly incorporated in the muscle setup held together by the rigid agarose gel, while the particles freely suspended in the medium were able to swell freely. The swelling of PLGA was reported in many studies to control the drug release from PLGA dosage forms (19, 24, 44–46) and the more hydrophilic acid-terminated grades swell faster than the ester-terminated (47, 48). This effect was also observed for the risperidone-loaded Resomer® 505 formulation in the later stages of advanced polymer degradation and hydration. The swelling might be similarly limited also in the tight interstitial space upon *in vivo* administration.

There was a lower amount of lidocaine and risperidone released from the ethyl cellulose particles on the first day in the muscle setup than in the pure medium, which can be attributed to the additional diffusion step of the released drug through the muscle tissue. This retarding effect was also observed in the case of 505 PLGA grade with the higher flurbiprofen loading (**≈**15%); however, not in the case of the low drug loadings. This additional retarding step is, therefore, apparently more prominent when a high initial drug amount is released (burst release). A similar effect was observed in a study by Andhariya et al. *in vivo*, when upon i.m. administration of leuprolide acetate loaded PLGA microspheres, considerably lower drug plasma concentrations were detected in the initial stage compared with a high burst release *in vitro* (49). The authors attributed this effect to the additional step of diffusion and absorption of the peptide from the muscle tissue; yet the overall release was, nonetheless, faster *in vivo*. However, the diffusion of the macromolecules such as the leuprolide acetate might be much more impacted (hindered) than that of low-Mw drugs.

Lipids isolated from the muscle tissue were identified to accelerate the release of all three investigated drugs. Drug solubility is one of the crucial factors determining the release rate from polymeric matrices (22). The tissue lipids increased the solubility of our model drugs and this effect could not only explain the accelerated release in our experiments but also the generally reported faster release rates *in vivo*. The presence of the fatty acids, lipid membranes, and other components of the ECM in the tissue might make the surroundings of the implanted formulation more lipophilic compared with the standard phosphate buffer, hence favor the release by increased drug solubility as observed *in vitro* in this study. Higher lidocaine solubility and its faster release from a gel formulation in human peritoneal fluid than in phosphate buffer have been reported and were attributed to the presence of physiological surfactants (50). However, the presence of lipids in the release medium did not affect the release from the acid-terminated PLGA grades in our experiments, but the higher solubility should unspecifically increase the release rate from all grades. Therefore, the accelerated release does not appear to be solely explained simply by a general solubility increase in the surrounding medium.

The lipids/fatty acids will tend to adsorb and accumulate on the hydrophobic surface of the microspheres. Adsorption of lipids on the hydrophobic surface of the implanted materials has been already shown to occur *in vivo* (51–53). The partitioning of a hydrophobic drug from the “very hydrophobic” PLGA/ethyl cellulose matrix into the “very hydrophilic” release medium (in other words, the release) might be made more favorable by this hydrophobic “intermediate compartment” of the lipid layer adsorbed on the surface.

Alternatively, the lipids and fatty acids can penetrate inside the polymer matrix (54) and enhance absorption of the release medium into the core of the matrix by osmotic effect and/or by counteracting the repulsive forces between the hydrophobic polymer chains and water molecules. Absorption of lipids into implanted silicone prosthetics (29, 55–57) and poly(glycolic acid) sutures (28) with impact on their structural and mechanical properties has been already documented earlier; therefore, the drug-loaded implants can be similarly affected with a direct consequence on drug release rate. Lipids co-encapsulated with risperidone in PLGA microspheres have been intentionally used to modify (accelerate) risperidone release (58).

The lipids in medium had an accelerating effect on the drug release, despite that the sink conditions in the lipid-free medium were already provided. Although the lipids emulsified in the release medium create a biphasic system, this is the important difference from the biphasic dissolution testing of oral dosage forms, where non-sink conditions exist in the aqueous phase and the drug partitioning into the organic phase prevents the drug saturation in the aqueous phase (5). However, in the microenvironment inside of the polymer matrix, the sink conditions may not be maintained, as demonstrated in a study by Siepmann et al. (59). The authors found that despite the sink conditions in the surrounding release medium, non-sink conditions inside of hypromellose matrix might exist even for freely soluble drugs. The authors further concluded that if such effect was observed even for relatively hydrophilic hypromellose matrices in a highly hydrated and swollen state, the effect might be even stronger for more hydrophobic matrices containing much less water (60). In a further study by the same research group, an equivalent situation was observed also in the case of PLGA extrudates (45). Correspondingly, the lipids and fatty acids in our experiments might have acted on increasing of the solubility in the microenvironment inside the microspheres upon their absorption into the matrix, despite sufficient sink conditions in the surrounding medium were provided already in the lipid-free medium. The acid-terminated PLGA grades being more hydrophilic allow faster water penetration inside the matrix and swelling; hence, the local drug saturation inside their matrix might not have been the rate-determining factor—possibly explaining why the lipids accelerated the release only from the ester-terminated PLGA and the ethyl cellulose microspheres.

Possible acceleration of the hydrolytic degradation of the PLGA is unlikely since in the experiments with the (non-degradable) ethyl cellulose, we have also observed faster release in the lipid-containing medium and in the muscle setup. The diffusion-controlled release from the ethyl cellulose matrices is characterized by decreasing rate with the decreasing concentration gradient (first-order kinetics) and the lipids seemed to partially prevent this rate deceleration—leading to overall faster release. On the other hand, in the case of the Resomer® 505, the effect of the lipids seemed to be on shifting of the onset of the phase of rapid release to sooner time points and to shortening of the lag phase.

Despite the exact mechanisms are yet to be elucidated, the interactions with lipids could, nevertheless, in some cases provide an explanation to the often reported faster drug release *in vivo* than in simple buffers *in vitro*.

The muscle tissue-based release methods shown here represent the first attempts towards the mimicking of the intramuscular conditions and are surely not without limitations. Clearly, the characteristic morphological structure of the native muscle fibers is not retained in the pulverized freeze-dried tissue. The limitation of swelling had an impact on the drug release, but this was determined by the strength of the agarose gel and may not resemble the actual physical properties of muscle tissue. Also, the lipids in tissue are not present freely as when emulsified in release medium and are mainly organized as lipid droplets inside of adipocytes, as bilayer in cell membranes, or the fatty acids bound on albumin (61, 62). However, displacement from this physiological distribution could still occur when the partitioning towards the implant surface is thermodynamically favorable or due to mechanical impact on the tissue caused by implant administration. The natural lipids are accompanied with a risk of chemical instability during the long testing period which may also lead to a change in the properties of the release medium in time. However, frequent replacement with medium with fresh lipids can overcome this issue.

## Conclusion

A muscle tissue homogenate-based drug release testing method was suggested here to closer mimic the intramuscular administration of depot microspheres. In addition to the novel biorelevant design, additional essential parameters of pharmaceutical quality control were considered in the development, such as long-term stability, reproducibility, low costs, convenience of sampling, and ease of preparation. From the diverse biological components of muscle tissue, particularly, the lipid components affected the drug release from the microspheres and these findings represent a previously unknown factor influencing the drug release from parenteral depot microspheres. The accelerating effect of the lipids was observed for all drugs tested and across chemically different polymers (PLGA and ethyl cellulose) but differed within different grades of the same polymer (acid- vs. ester-terminated PLGA). Although these experiments cannot conclusively tell whether the same interactions will happen *in vivo*, the results of this study strongly suggest the biological lipids as one of the important factors responsible for the differences between *in vitro* and *in vivo* release. Further studies will be necessary on investigation whether the whole mixture of the lipid extract or a single component is responsible for the effect and if the same effect can be achieved by a single triglyceride/fatty acid as a pure standardized substance. Our findings can provide basis for further more complex biorelevant models.

## Supplementary Information

ESM 1 (DOCX 14 kb)

## References

[1] Martinez MN, Rathbone MJ, Burgess D, Huynh M. Breakout session summary from AAPS/CRS joint workshop on critical variables in the in vitro and in vivo performance of parenteral sustained release products. J Control Release. 2010;142(1):2–7. 10.1016/j.jconrel.2009.09.028. 10.1016/j.jconrel.2009.09.028 19808069

[2] Martinez M, Rathbone M, Burgess D, Huynh M. In vitro and in vivo considerations associated with parenteral sustained release products: A review based upon information presented and points expressed at the 2007 Controlled Release Society Annual Meeting. J Control Release. 2008;129(2):79–87. 10.1016/j.jconrel.2008.04.004. 10.1016/j.jconrel.2008.04.004 18514351

[3] D’Souza SS, De Luca PP (2006). Methods to assess in vitro drug release from injectable polymeric particulate systems. Pharm Res.

[4] Seidlitz A, Weitschies W (2012). In-vitro dissolution methods for controlled release parenterals and their applicability to drug-eluting stent testing. J Pharm Pharmacol.

[5] Pestieau A, Evrard B. In vitro biphasic dissolution tests and their suitability for establishing in vitro-in vivo correlations: A historical review. Eur J Pharm Sci. 2017;102:203–19. 10.1016/j.ejps.2017.03.019. 10.1016/j.ejps.2017.03.019 28315463

[6] Fotaki N, Vertzoni M (2010). Biorelevant dissolution methods and their applications in in vitro-in vivo correlations for oral formulations. Open Drug Deliv J.

[7] Klein S. The use of biorelevant dissolution media to forecast the in vivo performance of a drug. AAPS J. 2010 [cited 2020 Aug 10]. p. 397–406. Available from: /pmc/articles/PMC2895438/?report=abstract 10.1208/s12248-010-9203-3 PMC2895438 20458565

[8] Iyer SS, Barr WH, Karnes HT (2007). Characterization of a potential medium for “biorelevant” in vitro release testing of a naltrexone implant, employing a validated stability-indicating HPLC method. J Pharm Biomed Anal.

[9] Kinnunen HM, Sharma V, Contreras-Rojas LR, Yu Y, Alleman C, Sreedhara A (2015). A novel in vitro method to model the fate of subcutaneously administered biopharmaceuticals and associated formulation components. J Control Release.

[10] Magri G, Selmin F, Cilurzo F, Fotaki N (2019). Biorelevant release testing of biodegradable microspheres intended for intra-articular administration. Eur J Pharm Biopharm.

[11] Sterner B, Harms M, Weigandt M, Windbergs M, Lehr CM (2014). Crystal suspensions of poorly soluble peptides for intra-articular application: A novel approach for biorelevant assessment of their in vitro release. Int J Pharm.

[12] Smith AM, Fleming L, Wudebwe U, Bowen J, Grover LM (2014). Development of a synovial fluid analogue with bio-relevant rheology for wear testing of orthopaedic implants. J Mech Behav Biomed Mater.

[13] Sun Y, Jensen H, Petersen NJ, Larsen SW, Østergaard J (2018). Concomitant monitoring of implant formation and drug release of in situ forming poly (lactide-co-glycolide acid) implants in a hydrogel matrix mimicking the subcutis using UV–vis imaging. J Pharm Biomed Anal.

[14] Ye F, Larsen SW, Yaghmur A, Jensen H, Larsen C, Østergaard J (2012). Drug release into hydrogel-based subcutaneous surrogates studied by UV imaging. J Pharm Biomed Anal.

[15] Klose D, Azaroual N, Siepmann F, Vermeersch G, Siepmann J (2009). Towards more realistic in vitro release measurement techniques for biodegradable microparticles. Pharm Res.

[16] Brandl F, Kastner F, Gschwind RM, Blunk T, Teßmar J, Göpferich A (2010). Hydrogel-based drug delivery systems: Comparison of drug diffusivity and release kinetics. J Control Release.

[17] Jensen SS, Jensen H, Møller EH, Cornett C, Siepmann F, Siepmann J (2016). In vitro release studies of insulin from lipid implants in solution and in a hydrogel matrix mimicking the subcutis. Eur J Pharm Sci.

[18] Allababidi S, Shah JC (1998). Kinetics and Mechanism of Release from Glyceryl Monostearate-Based Implants: Evaluation of Release in a Gel Simulating in Vivo Implantation. J Pharm Sci.

[19] Kozak J, Rabiskova M, Lamprecht A (2020). In-vitro drug release testing of parenteral formulations via an agarose gel envelope to closer mimic tissue firmness. Int J Pharm.

[20] Kinnunen HM, Mrsny RJ (2014). Improving the outcomes of biopharmaceutical delivery via the subcutaneous route by understanding the chemical, physical and physiological properties of the subcutaneous injection site. J Control Release.

[21] Zolnik BS, Burgess DJ (2008). Evaluation of in vivo-in vitro release of dexamethasone from PLGA microspheres. J Control Release.

[22] Fredenberg S, Wahlgren M, Reslow M, Axelsson A (2011). The mechanisms of drug release in poly(lactic-co-glycolic acid)-based drug delivery systems - a review. Int J Pharm.

[23] Anderson JM, Shive MS. Biodegradation and biocompatibility of PLA and PLGA microspheres. Adv Drug Deliv Rev. 2012;64:72–82. 10.1016/j.addr.2012.09.004. 10.1016/s0169-409x(97)00048-3 10837562

[24] Fang Y, Zhang N, Li Q, Chen J, Xiong S, Pan W (2019). Characterizing the release mechanism of donepezil-loaded PLGA microspheres in vitro and in vivo. J Drug Deliv Sci Technol.

[25] Doty AC, Weinstein DG, Hirota K, Olsen KF, Ackermann R, Wang Y (2017). Mechanisms of in vivo release of triamcinolone acetonide from PLGA microspheres. J Control Release.

[26] Rawat A, Bhardwaj U, Burgess DJ (2012). Comparison of in vitro–in vivo release of Risperdal® Consta® microspheres. Int J Pharm.

[27] Alexis F (2005). Factors affecting the degradation and drug-release mechanism of poly(lactic acid) and poly[(lactic acid)-co-(glycolic acid)]. Polym Int.

[28] Sharma CP, Williams DF. The effects of lipids on the mechanical properties of polyglycolic acid sutures. Eng Med. 1981;10(1):8–10. 10.1243/EMED_JOUR_1981_010_005_02.

[29] Cuddihy EF, Moacanin J, Roschke EJ, Harrison EC. In vivo degradation of silicone rubber poppets in prosthetic heart valves. J Biomed Mater Res. 1976;10(3):471–81. 10.1002/jbm.820100314. 10.1002/jbm.820100314 1270461

[30] Abd E, Yousef SA, Pastore MN, Telaprolu K, Mohammed YH, Namjoshi S, et al. Skin models for the testing of transdermal drugs. Clin Pharmacol Adv Appl. 2016;8:163–76. 10.2147/CPAA.S64788. 10.2147/CPAA.S64788 PMC5076797 27799831

[31] Jacobi U, Kaiser M, Toll R, Mangelsdorf S, Audring H, Otberg N (2007). Porcine ear skin: An in vitro model for human skin. Skin Res Technol.

[32] Chung B, Lee H, Choi M, Seo KY, Kim EK, Kim TI (2018). Preloaded and non-preloaded intraocular lens delivery system and characteristics: Human and porcine eyes trial. Int J Ophthalmol.

[33] Shelley H, Rodriguez-Galarza RM, Duran SH, Abarca EM, Babu RJ (2018). In Situ Gel Formulation for Enhanced Ocular Delivery of Nepafenac. J Pharm Sci.

[34] Loch C, Zakelj S, Kristl A, Nagel S, Guthoff R, Weitschies W, Seidlitz A (2012). Determination of permeability coefficients of ophthalmic drugs through different layers of porcine, rabbit and bovine eyes. Eur J Pharm Sci.

[35] Shen J, Choi S, Qu W, Wang Y, Burgess DJ (2015). In vitro-in vivo correlation of parenteral risperidone polymeric microspheres. J Control Release.

[36] Semmling B, Nagel S, Sternberg K, Weitschies W, Seidlitz A (2013). Long-term stable hydrogels for biorelevant dissolution testing of drug-eluting stents. J Pharm Technol Drug Res.

[37] Liang S, Xu J, Weng L, Dai H, Zhang X, Zhang L (2006). Protein diffusion in agarose hydrogel in situ measured by improved refractive index method. J Control Release.

[38] Sirianni RW, Kremer J, Guler I, Chen Y-L, Keeley FW, Saltzman WM (2008). Effect of extracellular matrix elements on the transport of paclitaxel through an arterial wall tissue mimic. Biomacromolecules.

[39] Leung DH, Kapoor Y, Alleyne C, Walsh E, Leithead A, Habulihaz B (2017). Development of a convenient in vitro gel diffusion model for predicting the in vivo performance of subcutaneous parenteral formulations of large and small molecules. AAPS PharmSciTech.

[40] Kurtz A, Pape H-C, Silbernagl S, Bondke Persson A, Brenner B, Burckhardt G, et al. 13 Wärmehaushalt und Temperaturregulation. Physiologie. 2018. 10.1055/b-006-149284.

[41] Routledge PA, Barchowsky A, Blornsson TD, Kitchell BB, Shand DG (1980). Lidocaine plasma protein binding. Clin Pharmacol Ther.

[42] Szpunar GJ, Albert KS, Wagner JG (1989). Pharmacokinetics of flurbiprofen in man II. Plasma protein binding. Res Commun Chem Pathol Pharmacol.

[43] Aarons L, Khan AZ, Grennan DM, Alam-Siddiqi M (1985). The binding of flurbiprofen to plasma proteins. J Pharm Pharmacol.

[44] Gasmi H, Willart JF, Danede F, Hamoudi MC, Siepmann J, Siepmann F. Importance of PLGA microparticle swelling for the control of prilocaine release. J Drug Deliv Sci Technol. 2015;30:123–32. 10.1016/j.jddst.2015.10.009.

[45] Bode C, Kranz H, Fivez A, Siepmann F, Siepmann J (2019). Often neglected: PLGA/PLA swelling orchestrates drug release: HME implants. J Control Release.

[46] Gasmi H, Danede F, Siepmann J, Siepmann F (2015). Does PLGA microparticle swelling control drug release? New insight based on single particle swelling studies. J Control Release.

[47] Samadi N, Abbadessa A, Di Stefano A, Van Nostrum CF, Vermonden T, Rahimian S, et al. The effect of lauryl capping group on protein release and degradation of poly(d,l-lactic-co-glycolic acid) particles. J Control Release. 2013;172(2):436–43. 10.1016/j.jconrel.2013.05.034. 10.1016/j.jconrel.2013.05.034 23751568

[48] Holgado MA, Arias JL, Cózar MJ, Alvarez-Fuentes J, Gañán-Calvo AM, Fernández-Arévalo M (2008). Synthesis of lidocaine-loaded PLGA microparticles by flow focusing. Effects on drug loading and release properties. Int J Pharm.

[49] Andhariya JV, Jog R, Shen J, Choi S, Wang Y, Zou Y (2019). Development of Level A in vitro-in vivo correlations for peptide loaded PLGA microspheres. J Control Release.

[50] Bhusal P, Rahiri JL, Sua B, McDonald JE, Bansal M, Hanning S (2018). Comparing human peritoneal fluid and phosphate-buffered saline for drug delivery: do we need bio-relevant media?. Drug Deliv Transl Res.

[51] Fröhlich SM, Eilenberg M, Svirkova A, Grasl C, Liska R, Bergmeister H (2015). Mass spectrometric imaging of in vivo protein and lipid adsorption on biodegradable vascular replacement systems. Analyst.

[52] Choi J, Lee BS, Park K, Han DK, Park KD, Kim YH. Beneficial effect of sulfonated peografted polyurethanes on calcification and lipid adsorption of vascular implants. Macromol Res. 2010;18:1133–6. 10.1007/s13233-010-1112-x.

[53] Fröhlich SM, Archodoulaki V-M, Allmaier G, Marchetti-Deschmann M (2014). MALDI-TOF Mass Spectrometry Imaging Reveals Molecular Level Changes in Ultrahigh Molecular Weight Polyethylene Joint Implants in Correlation with Lipid Adsorption. Anal Chem.

[54] Menei P, Daniel V, Montero-Menei C, Brouillard M, Pouplard-Barthelaix A, Benoit JP. Biodegradation and brain tissue reaction to poly(D,L-lactide-co-glycolide) microspheres. Biomaterials. 1993;14(6):470–8. 10.1016/0142-9612(93)90151-Q. 10.1016/0142-9612(93)90151-q 8507795

[55] Carmen R, Mutha SC (1972). Lipid absorption by silicone heart valve poppets—in-vivo and in-vitro results. J Biomed Mater Res.

[56] Carmen R, Kahn P (1968). In vitro testing of silicone rubber heart-valve poppets for lipid absorption. J Biomed Mater Res.

[57] Swanson JW, Lebeau JE (1974). The effect of implantation on the physical properties of silicone rubber. J Biomed Mater Res.

[58] Janich C, Friedmann A, Martins de Souza e Silva J, Santos de Oliveira C, de Souza LE, Rujescu D (2019). Risperidone-Loaded PLGA–Lipid Particles with Improved Release Kinetics: Manufacturing and Detailed Characterization by Electron Microscopy and Nano-CT. Pharmaceutics.

[59] Siepmann F, Karrout Y, Gehrke M, Penz FK, Siepmann J. Limited drug solubility can be decisive even for freely soluble drugs in highly swollen matrix tablets. Int J Pharm. 2017;526(1–2):280–90. 10.1016/j.ijpharm.2017.05.001. 10.1016/j.ijpharm.2017.05.001 28487190

[60] Siepmann J, Siepmann F. Sink conditions do not guarantee the absence of saturation effects. Int J Pharm. 2020;577 [cited 2020 Sep 9]. Available from: https://pubmed.ncbi.nlm.nih.gov/31917299/. 10.1016/j.ijpharm.2019.119009 31917299

[61] van der Vusse GJ. Albumin as fatty acid transporter. Drug Metab Pharmacokinet. 2009;24(4):300–7. 10.2133/dmpk.24.300. 10.2133/dmpk.24.300 19745557

[62] Eriksson U, Falkevall A. Visualizing Fatty Acid Flux. 2018;27:1161–2. 10.1016/j.cmet.2018.05.017. 10.1016/j.cmet.2018.05.017 29874562

