# Supplementary material for: Muscle Tissue as a Surrogate for In Vitro Drug Release Testing of Parenteral Depot Microspheres
Source: AAPS PharmSciTech. 2021 Mar 29;22(3):119. doi: 10.1208/s12249-021-01965-4 (PMC8007510; doi:10.1208/s12249-021-01965-4)
Supplement: Supplementary file 1 — (DOCX 14 kb) [file 12249_2021_1965_MOESM1_ESM.docx]

**HPLC analysis validation**

A) flurbiprofen

Linearity range: 0.5 µg/ml – 50 µg/ml

r^2^= 0.9998

LOQ = 0.075 µg/ml

B) lidocaine

Linearity range: 0.5 µg/ml – 50 µg/ml

r^2^= 0.9997

LOQ = 0.120 µg/ml

C) risperidone

Linearity range: 0.5 µg/ml – 50 µg/ml

r^2^= 0.9998

LOQ = 0.085 µg/ml
